# Estimating ^225^*Ac* yield in thorium metal targets

**DOI:** 10.1038/s41598-023-41687-0

**Published:** 2023-09-22

**Authors:** M. Rahmani, D. M. Martinez

**Affiliations:** Department of Chemical and Biological Engineering, University of British Columbia, Vancouver, BC V6T 1Z4 Canada

**Keywords:** Biomedical engineering, Applied mathematics

## Abstract

In this work we estimate the yield of the radioisotope $^{225}Ac$ in a thorium metal target geometry similar to that described by Roberston et al.. We do so in three different yet complimentary studies. In the first study, we pose a three-way coupled time-dependent model describing beam position, temperature field, and local growth of the activity of the radioisotope and solve this numerically. In the second study, we present an analytical solution of the model equations for a generalized solid target in the “beam-thin” limit, i.e. where only a small fraction of the incoming energy of the proton beam is deposited into the thorium material. In the third study, we use the insight gained from the analytical solution and describe an operating strategy to maximize yield by modulating the beam flux temporally.

## Introduction

**Figure 1:**
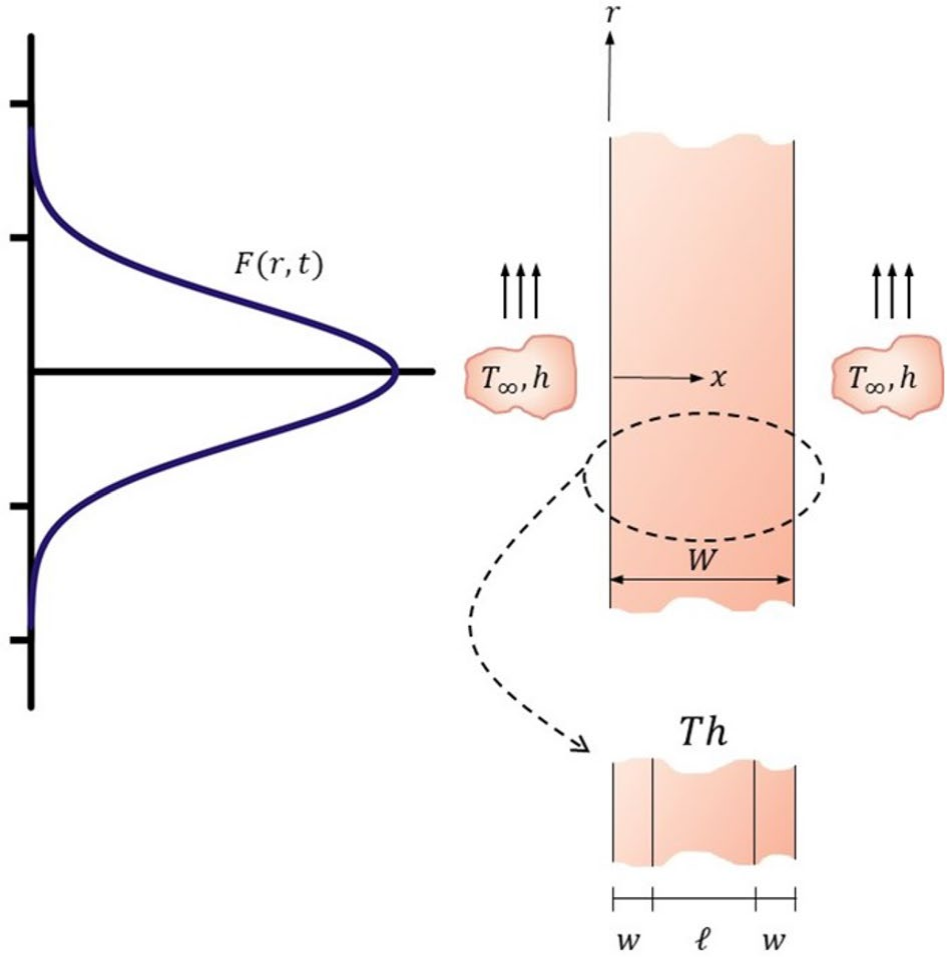
Schematic of the model geometry geometry considered. The beam enters the cylindrical target from the left. The Gaussian curve, drawn in blue, represents the distribution in flux *F*(*r*, *t*). The target package is of width *W* and is a composite structure comprised of three layers as shown in the inset. The central body is Thorium (*Th*) and it is within this domain that the reaction to produce $^{225}Ac$ occurs.

Understanding the production of radionuclides in solid targets has a rich history dating back to Krasnov^1^. Radioisotopes are created by the interplay between charged hadrons, flowing at a flux of *F*(*r*, *t*) ($1/{\rm m}^{2}s$) through a material of density $\rho (x)$ ($\text {atom}/{\rm m}^3$), see Fig. 1. Interaction between the hadron with matter causes, beneficially, the formation of an radioisotope at a rate proportional to the nuclear cross-section $\sigma (E)$ (${\rm m}^2$), which is a function of the local energy of the beam *E*(*x*) (*J*). This interaction also causes significant internal heat generation as the hadron slows to its resting state. Krasnov^1^ advances that the rate of production of an isotope *N* ($\text {atom}/{\rm m}^3$) is a delicate balance between isotope-creation and natural decay, which commences immediately following formation of the radioactive species. Expressed mathematically, he advances a balance of the form

1 $$\begin{aligned} \frac{\partial N}{\partial t} = F\rho \sigma - \lambda N. \end{aligned}$$

As *N* is related to activity $A_c$ through

2 $$\begin{aligned} A_c = \lambda \int _V \,N \,\textrm{d}V. \end{aligned}$$

Krasnov’s forumation may be expressed as

3 $$\begin{aligned} \frac{1}{\lambda }\frac{{\textrm{d}} A_c}{{\textrm{d}} t} = \int _V F\rho \sigma dV - A_c \end{aligned}$$

where $\lambda$ is the decay constant and *V* is the volume of the target. The utility of these expressions have been demonstrated over the years for a wide range of media and complex structures^2^. More importantly, these relationships admit to maximum or “saturated” activity, $A_c^{max}$, defined when

4 $$\begin{aligned} \frac{\textrm{d}A_c}{\textrm{d}t}\,=\,0\quad \quad \longrightarrow \quad \quad A_c^{max} = \int _V F\rho \sigma dV \end{aligned}$$

Achieving this maximum is crucial in order to optimize radioisotope production rate in a clinical setting.

As $A_c^{max}$ is proportional to the irradiation flux *F*, heat transfer constrains the permissible range of the beam flux, as solid target materials have poor thermal conductivity, low melting points and fail due to thermal stress. Several groups have advanced models of the thermal performance by balancing the heat generated by the beam with that removed by convection^3,4, 5^. In perhaps the most sophisticated version of this style of modeling, O’Brien et al.^4,6^ utilized Monte-Carlo methods to estimate the (time-dependent) beam position and energy transfer to the solid target and coupled these to a RANS (Reynolds-averaged Navier-Stokes) formulation to understand the convective mechanism. Whilst powerful, these approaches are computationally expensive, limiting their utility as a design tool.

The motivation behind this work stems from a rapidly developing therapy for late-stage cancer, i.e. targeted alpha radiopharmaceuticals using $^{225}Ac$ produced from Thorium metal targets; availability of this radioisotope is critical to advance therapy. A secondary motivation is the need to develop a simple computational approach to optimize production under the constraint of internal heating. We examine this in three separate sections. In the first section, we pose a three-way coupling between the temperature field, the position of the beam, governed by the Bethe equation, and isotope production and solve this system numerically. We demonstrate a weak-coupling between the temperature field and beam position and for the specific case of $^{225}$Ac, energy deposition is found to be nearly uniform throughout the volume of the thorium. In the second section, we gain insight into the full-numerical solution by developing an asymptotic solution. Here we generalize Kransov’s study and advance estimates of the temperature and activity profiles in the limit of small energy deposition, i.e. when operated in the “beam-thin” limit, and when the beam has radial variations in its flux. This is the limit in which $^{225}$Ac targets are operated. We extend this work and examine the effect of time-dependency created through beam-modulation and demonstrate an operating strategy where we are able to maximize the yield without risk of overheating. In the final section, we demonstrate the utility of our numerical model and apply this to a vastly different target system, the production of $^{99m}$Tc, and are able to reproduce literature results.

## Target model

We use a target geometry similar to that reported by Roberston et al.^3^ for the production of $^{225}Ac$. The target is a composite material with a Thorium foil, of thickness $\ell$, as the central body, sandwiched between fore-aft inconell foils, of thickness *w*, which serves for mechanical support and environmental protection^3^. The cylindrical solid-target is of radius *R* and total width of $W=2w+\ell$. The target is considered to be thin such that $W/R\ll 1$.

At $t\le 0$, the target has an uniform initial temperature $T_\infty$, which is equivalent to the temperature of the ambient flow field. At $t > 0$, a proton beam is introduced and is distributed according to

5a $$\begin{aligned} F(r,t) = F_og(t) e^{-(r/\omega )^2} \end{aligned}$$

where $F_o$ is maximum beam flux on the central axis; $\omega ^2$ is known as the width parameter^5^; and *g*(*t*) is a function describing the time-variations of the current representing either start-up or modulation^4^. As the beam intensity is usually reported as a current *I* ($\upmu$A), the particle flux is related to *I* using^1^

5b $$\begin{aligned} 2\pi \int _0^R F_og(t) e^{-(r/\omega )^2}r\,\textrm{d}r=6.24\times 10^{18}\,I \end{aligned}$$

for particles with a relative charge of unity^1^, implying that $F_o$ is related to the current using

5c $$\begin{aligned} F_o = \frac{6.24\times 10^{18}\,I}{\pi \omega ^2\left( 1-\exp (-(\omega /R)^2\right) } \end{aligned}$$

Following O’Brien et al.^4^, we consider the target to be heated internally by the beam and in each layer conservation of energy demands

6 $$\begin{aligned} \frac{1}{\alpha }\frac{\partial T}{\partial t} = \nabla ^2 T -\frac{F}{k}\frac{{\textrm{d}}E}{{\textrm{d}} x} \end{aligned}$$

where *T*(*x*, *r*, *t*) is the temperature; *E*(*x*) is the energy of the beam; *k* is the thermal conductivity of the material (W/mK); and $\alpha$ is the thermal diffusivity (${\rm m}^2/{\rm s}$). The negative sign on the source term is required as $\textrm{d}E/\textrm{d}x\le 0$. Using the notation reported by Grimes et al.^7^, we approximate *E* using the Bethe equation:

7 $$\begin{aligned} \frac{1}{m_p}\frac{\textrm{d}E}{\textrm{d}x}&=\gamma (u)^3 u\frac{d u}{d x} =-\frac{A}{ u^2}\left( \ln (\frac{ B u^2}{1-( u/c)^2})-( u/c)^2\right) \end{aligned}$$

were *u*(*x*) and *c* are the velocity of the particle and the speed of light, respectively ; $m_p$ is the mass of the particle; and $\gamma (u)$ is the Lorentz factor defined by

8 $$\begin{aligned} \gamma (u) = \frac{1}{\sqrt{(1-(u/c)^{2})}} \end{aligned}$$

Here, *A* (${\rm m}^3/{\rm s}^4$) and *B* $\left({\rm s}^2/{\rm m}^2 \right)$, are empirical constants as defined by Grimes et al.^7^. Finally, at left and right boundaries, $x=(0,W)$, we set the Robin condition

9 $$\begin{aligned} -k\nabla T\cdot \vec {n}=h(T - T_{\infty }) \end{aligned}$$

where $\vec n$ is the outward facing normal vector; *h* is the heat transfer coefficient (${\rm W}/{\rm m}^2 {\rm K}$); and $T_\infty$ is the temperature of the cooling water in contact with the target surface. A symmetry condition is enforced on the central axis ($r=0$), a no-flux condition at the outer periphery and the contact resistance between each layer of material is neglected.

Equations (1)–(9) represent the model equations and have been solved using two different methodologies. The model is posed over one domain with spatially varying material properties. In first methodology, we used a second-order accurate finite difference scheme for the spatial derivatives and advanced the temporal derivatives using an explict scheme. This problem was well-behaved as density varied weakly with temperature over the ranges considered, as the thermal expansion coefficient of materials used in this target are small. Because of this, we created a second numerical methodology and uncoupled the governing equations and increased the order of the approximations of the temporal derivatives. This allowed for a significant reduction in computational cost with a minimal reduction in accuracy. We note that we solved the Bethe equation using the numerical method outlined by Martinez et al.^8^ and with the results validated using the approximations presented by previous groups^7,8^.All numerical schemes were implemented in MATLAB.

**Figure 2:**
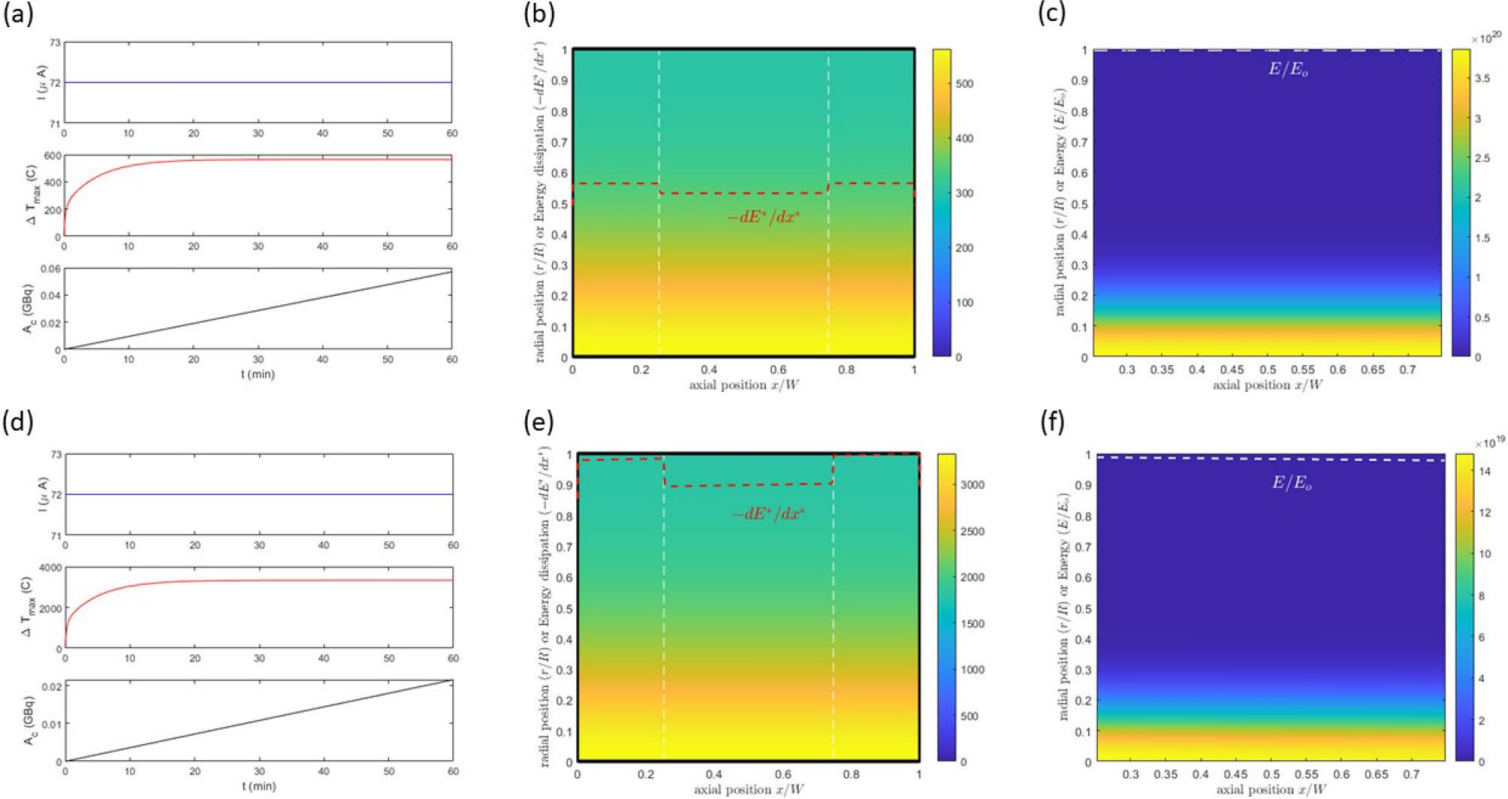
Two separate simulations conditions are presented in this experimental campaign. In (**a**–**c**), the simulations were conducted with a 451 MeV beam with the cross-section data given from the open literature^2^. More detail of the simulation conditions are given in Table 1. For energy levels greater than the range given from the literature data, the cross-section was assumed to constant at 19.4 mb. In detail, in (**a**), we display the current, maximum temperature and *Ac* as a function of time over the one hour simulation. The temperature profile and isotope distribution *N* are shown in (**b**) and (**c**). In (**d**–**f**), we display the second simulation. The simulations were conducted with a 100 MeV beam. In detail, in (**d**), we display the current, maximum temperature and *Ac* as a function of time over the one hour simulation. The temperature profile and isotope distribution *N* are shown in (**e**) and (**f**). In both simulations, we display the the energy profile $E/E_c$ as the white dashed lines in (**c**) and (**f**). In (**b**) and (**e**) we show the energy dissipation as the red dashed lines, and the position of each foil as the vertical white dashed lines. The energy dissipation $-dE/dx$ has been scaled so that it will fit on the graphs. The definition of the scaling will be given in the text subsequently. For all simulations $R=50$ mm, $w=0.1$ mm, $\ell =0.3$ mm, $\omega = 9$ mm and operated under a constant beam with $g(t)=1$. With a beam current of 72 $\upmu {\rm A}$, we determine $F_o= 1.77\times 10^{18}$ particles/m$^2$ s using Eq. (5c)

Two simulations are presented Fig. 2 demonstrating qualitatively the behavior at different initial beam energy levels. These simulations yield similar behaviors. The first observation that can be made is that over the one hour irradiation period, the activity increases linearly with time and (Fig. 2a–d). This is not unexpected as the one hour irradiation time is significantly smaller that the half-life of $^{225}Ac$, i.e. 9.92 days. We find similarity in the temperature distributions and unexpectedly, a significantly higher temperature was realized for the case with lower initial beam energy, as more energy is being deposited into the target under these conditions (Fig. 2b–e). This results from the non-linearity of the Bethe equation; this will be explored to a greater extent in the next section where we demonstrate that under these conditions the energy deposition gradient (*de*/*dx*) is proportional to the inverse of the initial speed of the beam. Uniquely, we would conclude that the target would melt in Fig. 2e but not in (b). In both simulations, we observe significant structure in the temperature and isotope concentration *N* in the radial direction, but very little in the axial direction. We argue that variations in the radial direction reflect the Gaussian distribution of the beam. In the axial direction, the activity is essentially constant as *E*(*x*) varies weakly in the direction of travel, i.e. the cross-sectional area is essentially constant. For the temperature profile, we argue that as the Biot number $(Bi\,\equiv \,hR/k)$ is small, we anticipate small axial gradients in the material in comparison to those found across the fluid boundary layer. The radial structure in the temperature profile is preserved as the material is thin and the dominant mechanism for cooling in in the axial direction. We find these observations quite unique, and robust over a wider range of conditions and explore their theoretical underpinnings in more detail in the next section.

**Table 1:** A listing of the parameters used for the simulations conducted in Fig. 2.

Foil	*A*	*B*	$C_p$	*k*	*h*
	$(\textrm{m}^3/\textrm{s}^4)$	$(\textrm{s}^2/\textrm{m}^2)$	$(\textrm{J}/\textrm{Kg}\,\textrm{K}$)	$(\textrm{W}/\textrm{m}\,\textrm{K})$	$(\textrm{W}/\textrm{m}^2 \textrm{K})$
Inconell	$1.1\times 10^{33}$	$4.4\times 10^{-14}$	444	15	50
Thorium	$1.2\times 10^{33}$	$1.3\times 10^{-14}$	440	54	50

The intermediary steps to calculate *A* and *B* are given as S2 of the Supplemental Material.

## Analysis

In this previous section, we advanced a model for $^{225}Ac$ targets where we observed that energy deposition (*dE*/*dx*) is nearly constant, and curiously, the maximum temperature increases with the lower incoming beam energy. We examine these observations in greater detail in order to (1) uncover the theoretical underpinnings and (2) to develop an operating strategy to maximize yield. To do so, we reduce the complexity of the target body, by considering constant material properties, in order to gain insight into the behavior of the system.

### Beam-energy distribution

In this sub-section, we approximate Eq. (7) for thin-target of uniform properties. We do so in two steps. We first examine the right-hand side of this equation and estimate *u*(*x*). With this in hand, we use this to approximate *E*(*x*), which is the left-hand side of the equation. We begin by assuming that the velocity profile *u*(*x*) obeys a function of the form

10 $$\begin{aligned} u = u_o( 1\,+\,\delta U_1(X)\,+\,\delta ^2 U_2(X)\,+\,\cdots) \hspace{3.in} \left( X=\frac{x}{W}\right) \end{aligned}$$

where $u_o$ is the velocity of the beam initially; $U_i$ are dimensionless functions of *O*(1), representing deviations from the initial velocity; and $\delta$ is a small parameter ($0\le \delta \ll 1$) whose value is *unknown* at this point. The parameter $\delta$ will be determined as part of the solution. Upon substitution of Eq. (10) into Eq. (7), and equating terms of equivalent powers in $\delta$, we find the following system of equations

11a $$\begin{aligned} \mathcal {O}(\delta ):&\hspace{0.5in}\frac{{\textrm{d}}U_1}{dX} \,=\, 1 \hspace{3.75in} \left( U_1(0)=0\right) \end{aligned}$$

11b $$\begin{aligned} \mathcal {O}\left( \delta ^2 \right) :&\hspace{0.5in}{\frac{{\textrm{d}} U_2}{\textrm{d}X}}\,=\,2U_1 \frac{\left( -1+ \left( 1 -{\varepsilon }^{2} \right) \ln \left( {\frac{\beta }{1-{\varepsilon }^{2}}} \right) \right) }{ \left( \left( \varepsilon ^{2}-1 \right) \ln \left( {\frac{\beta }{1- \varepsilon ^{2}}} \right) -{\varepsilon }^{4}+{\varepsilon }^{2} \right) }-\frac{1}{2}\left( 1+{\frac{3\varepsilon ^2}{1-\varepsilon ^2}} \right) {\frac{{\textrm{d}} U_1^2}{{{\textrm{d}}}X} \hspace{0.5in} \left( U_2(0)=0\right) } \end{aligned}$$

and are able to formally define

12 $$\begin{aligned} \delta = \frac{AW}{u_o^4} \left( \ln \left( \frac{\beta }{1-\varepsilon ^2}\right) - \varepsilon ^2\right) \left( \varepsilon ^2-1\right) ^{3/2} \hspace{0.5in} \left( \beta \equiv Bu_o^2,\,\,\varepsilon \equiv \frac{u_o}{c}\right) \end{aligned}$$

by equating $\delta$ to the leading order terms. With this, we estimate $\delta$ for the conditions simulated in Fig. 2 as $\delta \sim 10^{-3}$ at 100 MeV and $\delta \sim 10^{-4}$ at 451 MeV. When integrated

13a $$\begin{aligned} U_1\,&=\,X \end{aligned}$$

13b $$\begin{aligned} U_2 \,&=\,\frac{{X}^{2}}{2\,{\varepsilon }^{2}-2} \left( \frac{ -2\,{\varepsilon }^{4}-{\varepsilon }^{2}+3\,\ln \left( -{\frac{ \beta }{ \left( \varepsilon -1 \right) \left( \varepsilon +1 \right) }} \right) -2}{ \ln \left( -{\frac{\beta }{ \left( \varepsilon -1 \right) \left( \varepsilon +1 \right) }} \right) -{\varepsilon }^{ 2}} \right) \end{aligned}$$

We continue this analysis and examine the estimate of the energy distribution *E*(*x*). This estimate may be deduced directly from Eq. (7) as the velocity profile is known (cf. Eq. (13)). Formally, if we seek a solution of the form

14 $$\begin{aligned} E^{*}\equiv \frac{E(x)}{E_c}\,=\,1\,+\,\delta E_1(x)\,+\,\delta ^2 E_2(x)\,+\,\cdots \hspace{1in} \left( E_c\,\equiv \, m_p\gamma (u_o)^3 u_o^2\right) \end{aligned}$$

Eq. (7) reduces to

15a $$\begin{aligned} \frac{\textrm{d}E_1}{\textrm{d}X}\,&=\,\frac{\textrm{d}U_1}{\textrm{d}X}\longrightarrow \quad \quad E_1\,=\,U_1 \end{aligned}$$

15b $$\begin{aligned} \frac{\textrm{d}E_2}{\textrm{d}X} \,&=\,{\frac{ \left( 2 \,{\varepsilon }^{2}+1 \right) }{2(1-{\varepsilon }^{2})}}\frac{\textrm{d}U_{1}^{2}}{\textrm{d}X}+\frac{\textrm{d}U_2}{\textrm{d}X}\longrightarrow \quad \quad E_2\, = {\frac{ \left( 2 \,{\varepsilon }^{2}+1 \right) }{2(1-{\varepsilon }^{2})}}U_{1}^{2}\,+\,U_2 \end{aligned}$$

The significance of this analysis is two-fold. First, and perhaps most importantly, from this analysis we are able to clearly define the conditions for the “beam thin” operating conditions. We advance that “beam-thin” occurs when $\delta < 0.1$. In the non-relativistic limit, i.e. when $\varepsilon \rightarrow 0$, we advance a rule of thumb that beam-thin conditions exist when

16 $$\begin{aligned} W<\frac{0.1u_o^4}{A\ln \beta } \end{aligned}$$

Secondly, this result supports our observation that the energy dissipation *dE*/*dx* is *nearly* constant and that the energy profile *E*(*X*) decreases essentially linearly up to $\mathcal {O}(\delta ^2)$.

### Temperature distribution

At this point we turn our attention to the temperature distribution in the target. Our goal is to shed light onto the observation that the maximum temperature of the target *increases* with lower initial beam energy, see Fig. (2). Without loss of generality, we consider the steady state distribution, in the limit of small Biot number, i.e. $(Bi\equiv hW/k<1)$, and when the source term is weakly-dependent on radial position (${\bar{\omega }}\equiv \omega /R>1$). By doing so, the model becomes mathematically-tractable. Formally, if we integrate Eq. (6) over *x*-direction, and scale using

17a $$\begin{aligned} \theta = \frac{T-T_\infty }{T_c}\quad \& \quad {\bar{r}} = \frac{r}{R} \end{aligned}$$

where $T_c$ is a characteristic temperature defined as

17b $$\begin{aligned} \quad T_c \equiv -\delta \frac{F_o E_c}{kW}\,=\, \left[ \frac{m_p\,A\,I}{k\,u_o^2} \left( \ln (\frac{\beta }{1-\varepsilon ^2})- \varepsilon ^2\right) \right] \left[ \frac{6.24\times 10^{18}}{\pi \omega ^2\left( 1-\exp (-(\omega /R)^2\right) } \right] \end{aligned}$$

the governing equation reduces to

18 $$\begin{aligned} \frac{1}{{\bar{r}}}\frac{\textrm{d}}{\textrm{d}{\bar{r}}}\left( {\bar{r}}\frac{\textrm{d}\theta }{\textrm{d}{\bar{r}}}\right) -2Bi\theta \,+\,1\,-\,\left( \frac{{\bar{r}}}{\bar{\omega }}\right) ^2+\frac{1}{2}\left( \frac{{\bar{r}}}{{\bar{\omega }}}\right) ^4\,=\,0 \end{aligned}$$

with use of Eqs. (5c), (8), (9), (12), and (14). The Gaussian source term has been approximated by a Taylor series as ${\bar{\omega }} >1$.

**Figure 3:**
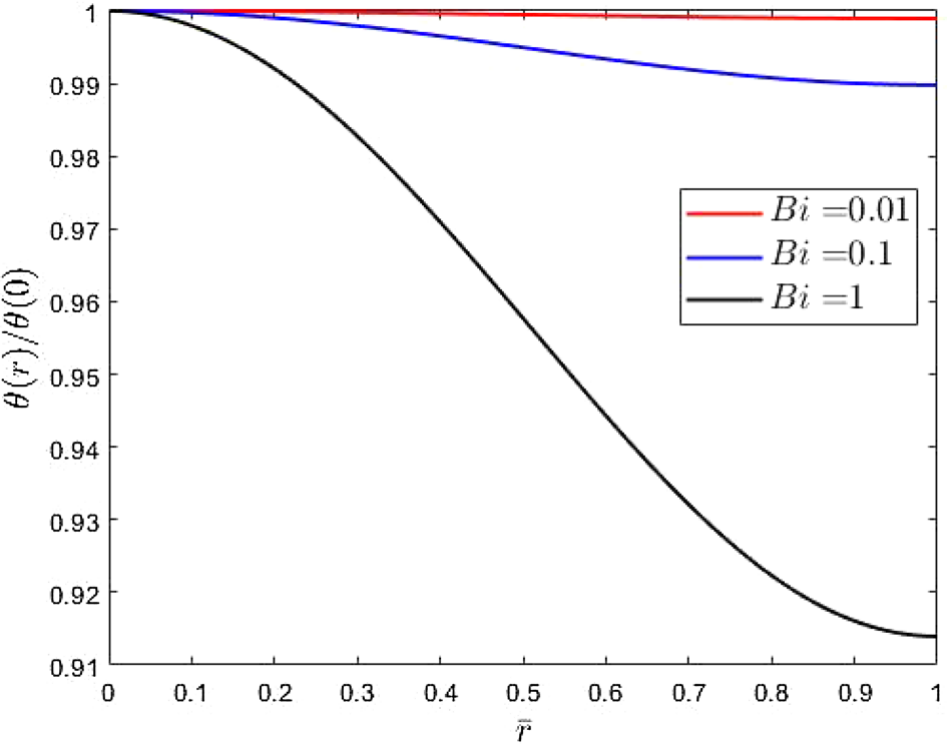
The normalized depth-averaged temperature distribution over the radius of a thin target body. The simulations were conducted with ${\bar{\omega }}=1$ at three different values of *Bi*.

When integrated, the temperature distribution is approximated as

19 $$\begin{aligned} \theta \,=\,+\frac{\left( Bi\,{{\bar{\omega }}}^{2}-Bi-4\right) }{{Bi}^{2}{{\bar{\omega }}}^{4} \sqrt{2\,Bi}} \frac{{ I}_{0}\left( \sqrt{2\,Bi}\,{\bar{r}}\right) }{{{ I}_{1}\left( \sqrt{2\,Bi}\right) }} +{\frac{16+ \left( 2\,{{\bar{\omega }}}^{4}-2\,{{\bar{\omega }}}^{2}{{\bar{r}}}^ {2}+{{\bar{r}}}^{4} \right) {Bi}^{2}+ \left( -4\,{{\bar{\omega }}}^{2}+8\,{{\bar{r}}}^{2} \right) Bi}{4\,{Bi}^{3}{{\bar{\omega }}}^{4}}} \end{aligned}$$

whose form is highlighted in Fig. 3. The intermediary steps in this integration are given in S1 of Supplemental Material. What is observed from this figure is that the peak temperature occurs at $r=0$ and decreases with increasing Biot numbers. With this, we estimate the maximum temperature rise as $\Delta T_{max}\equiv T-T_\infty =T_c\theta (0)$

20 $$\begin{aligned} \Delta T_{max} \,=\,T_c\left( +\frac{\left( Bi\,{{\bar{\omega }}}^{2}-Bi-4\right) }{{Bi}^{2}{{\bar{\omega }}}^{4} \sqrt{2\, Bi}} \frac{1}{ {{ I}_{1}\left( \sqrt{2\,Bi}\right) }} +{\frac{16+ \left( 2\,{{\bar{\omega }}}^{4}\right) {Bi}^{2}+ \left( -4\,{{\bar{\omega }}}^{2} \right) Bi}{4\,{Bi}^{3}{{\bar{\omega }}}^{4}}} \right) \end{aligned}$$

and argue that at a fixed Biot number (*Bi*) and width parameter ($\omega$)

21 $$\begin{aligned} \Delta T_{max}\,\propto T_c\quad \quad \longrightarrow \quad \quad \Delta T_{max}\propto \,\left[ \frac{m_p\,A\,I}{k\,u_o^2} \ln \,\beta \right] \left[ \frac{6.24\times 10^{18}}{\pi \omega ^2\left( 1-\exp (-(\omega /R)^2\right) } \right] \end{aligned}$$

in the non-relativistic limit, *i.e.* $\varepsilon \rightarrow 0$. This indicates that the maximum temperature increases linearly with current *I* but decreases inversely with energy of the beam $(u_o^2)$. This implies that the stopping power of proton increases with decreasing the energy. This supports our findings reported in Fig. 2, where larger temperatures were found at lower beam energy. We advance this as a rule of thumb for beam-thin targets.

### Activity distribution

In this section, we estimate the *N* and $A_c$ in the beam-thin limit. We are motivated to perform this analysis as Kransov’s widely cited work^1^ only considered the limit where the beam comes to rest in the material. If

22a $$\begin{aligned} N = N_c\left( N_o\,+\,\delta N_1\,+\,\cdots \right) \hspace{1in}\left( N_c=\frac{F_o\rho \sigma (E_c)}{\lambda }\right) \end{aligned}$$

and $\sigma (E)$ is expanded using a Taylor series in conjunction with Eqs. (14) and (15a), *i.e.*

22b $$\begin{aligned} \sigma (E)=\sigma ( E_c(1+\delta X))=\sigma (E_c)+ \delta X E_c\sigma (E_c)\frac{\textrm{d}\sigma }{\textrm{d}e}\bigg |_{E_c}\,+\,\cdots \end{aligned}$$

With this Eq. (1) reduces to

22c $$\begin{aligned} \mathcal {O}(1):&\hspace{0.5in}\frac{\partial N_o}{\partial {\bar{t}}} \,=\, e^{-\left( \frac{{\bar{r}} R}{\omega }\right) ^2}\,-\,N_o \hspace{1in} \left( N_o({\bar{r}},X, 0)=0\right) \end{aligned}$$

22d $$\begin{aligned} \mathcal {O}(\delta ):&\hspace{0.5in} \frac{\partial N_1}{\partial {\bar{t}}} \,=\, \sigma ^\prime e^{-\left( \frac{{\bar{r}} R}{\omega }\right) ^2}X \,-\,N_1 \hspace{0.75in} \left( N_1({\bar{r}},X, 0)=0\right) \end{aligned}$$

where the following dimensionless parameters have been defined for mathematical convenience

22e $$\begin{aligned} {\bar{t}}\,=\, t\lambda \hspace{0.5in} \sigma ^\prime \,=\,E_c\sigma (E_c)\frac{\textrm{d}\sigma }{\textrm{d}e}\bigg |_{E_c} \end{aligned}$$

These may be integrated to yield

$$\begin{aligned} N_1 =e^{-\left( \frac{\bar{\bar{r}} R}{\omega }\right) ^2} \left( 1-{\textrm{e}^{-{\bar{t}}}} \right) \hspace{0.5in} N_2 =\sigma ^\prime Xe^{-\left( \frac{\bar{\bar{r}} R}{\omega }\right) ^2} \left( 1-{\textrm{e}^{-{\bar{t}}}} \right) \end{aligned}$$

which in turn may be used to determine $A_c$ through use of Eq. (2), *i.e.*

23 $$\begin{aligned} A_c\,=\,A_c^{max}\left( 1-e^{-{\bar{t}}}\right) \hspace{1in} A_c^{max}=\sigma (E_c)\rho F_o\pi \omega ^2W\left( 1-e^{-(R/\omega )^2}\right) \end{aligned}$$

These results indicate that the isotope concentration *N* is essentially constant in the thickness direction but adopts a Gaussian distribution in the radial direction. Our results demonstrate, for the first time, that the estimate of the maximum activity is significantly different than that advanced by Kransov^1^. Crucially, the activity distribution is dictated primarily by the width parameter of the beam and the expression above properly accounts for this. We can recover Krasnov’s estimate when the beam becomes nearly uniform, i.e. in the limit $\omega \gg R$. These finding also give a strong hint that beam width may be an underlying reason for variation in recovered activity between batches or between different cyclotrons.

### Operating strategy

In the previous sub-section, we extended Krasnov’s analysis to a second limit, i.e. beam-thin conditions, and advance an asymptotic solution to the model equations. The solution reinforced the theoretical underpinnings of our observations given by the full-numerical solution. With these, we advance an operating strategy to maintain high beam current, and thus large yield, at a manageable temperature rise. We do so by modulating the beam temporally, in a series of on-off cycles. The length of the on-cycle ($t_h$) is dictated by the characteristic time of the target to heat-up to the set-point temperature; the length of the off-cycle ($t_c$) is dictated by the characteristic time for cooling. Critically, there will be no significant loss of activity if cooling time is much smaller than the half-life of the isotope, and we can achieve a desired activity by increasing the number of cycles, without fear of target failure. For this particular case as the half-life of *Ac* is significantly greater than $t_c$, there is very limited decay during the cooling period.

**Figure 4:**
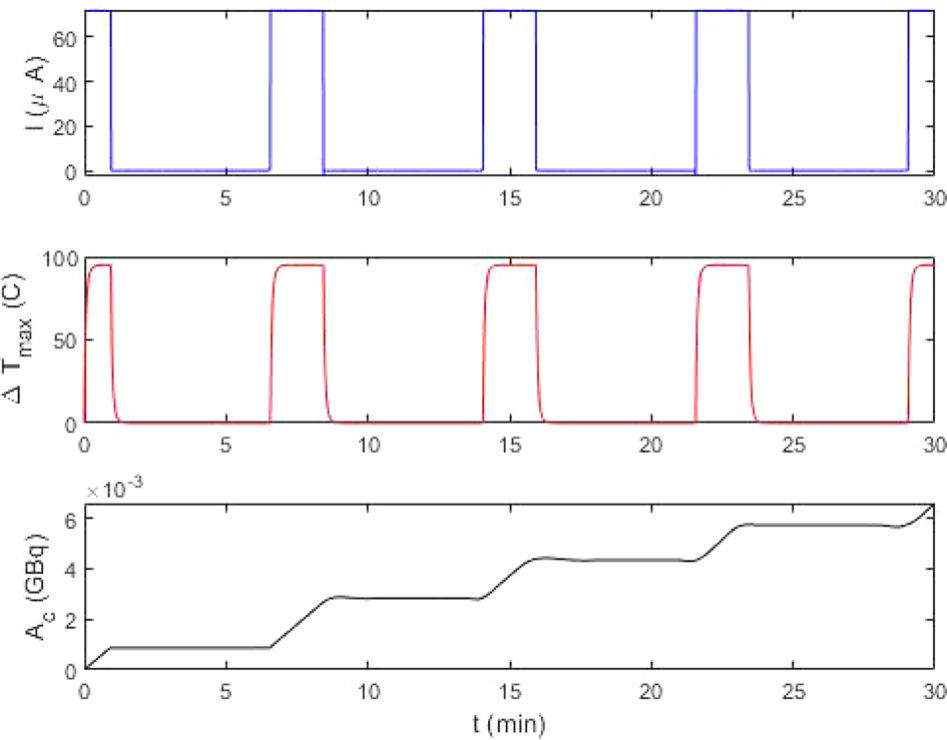
We display the current, maximum temperature and *Ac* as a function of time over the 30 min simulation. For this simulation, $R=50$ mm, $w=0.1$ mm, $\ell =0.3$ mm, $\omega = 9$ mm and operated with the beam being modulated over a 7 minute period ($t_h=2$ min and $t_c=5$ min. The density, heat capacity and thermal conductivity of all materials, and heat transfer coefficient are similar to that given by Roberston et al.^3^. With a beam current of 72 $A$, we determine $F_o= 1.77\times 10^{18}$ particles/m$^2$ s using Eq. (5c).

We investigated this numerically by setting the constraint of a temperature rise of $\Delta T^{max}=100$ C by cycling the beam current over a seven minute cycle (Fig. 4). Through optimization we discovered that there are multiple combinations to achieve this, depending upon peak current $F_o$ and initial energy input $E_o$. The results shown in this figure is just one potential solution and further constraints are required to identify optimal conditions.

## Discussion

**Figure 5:**
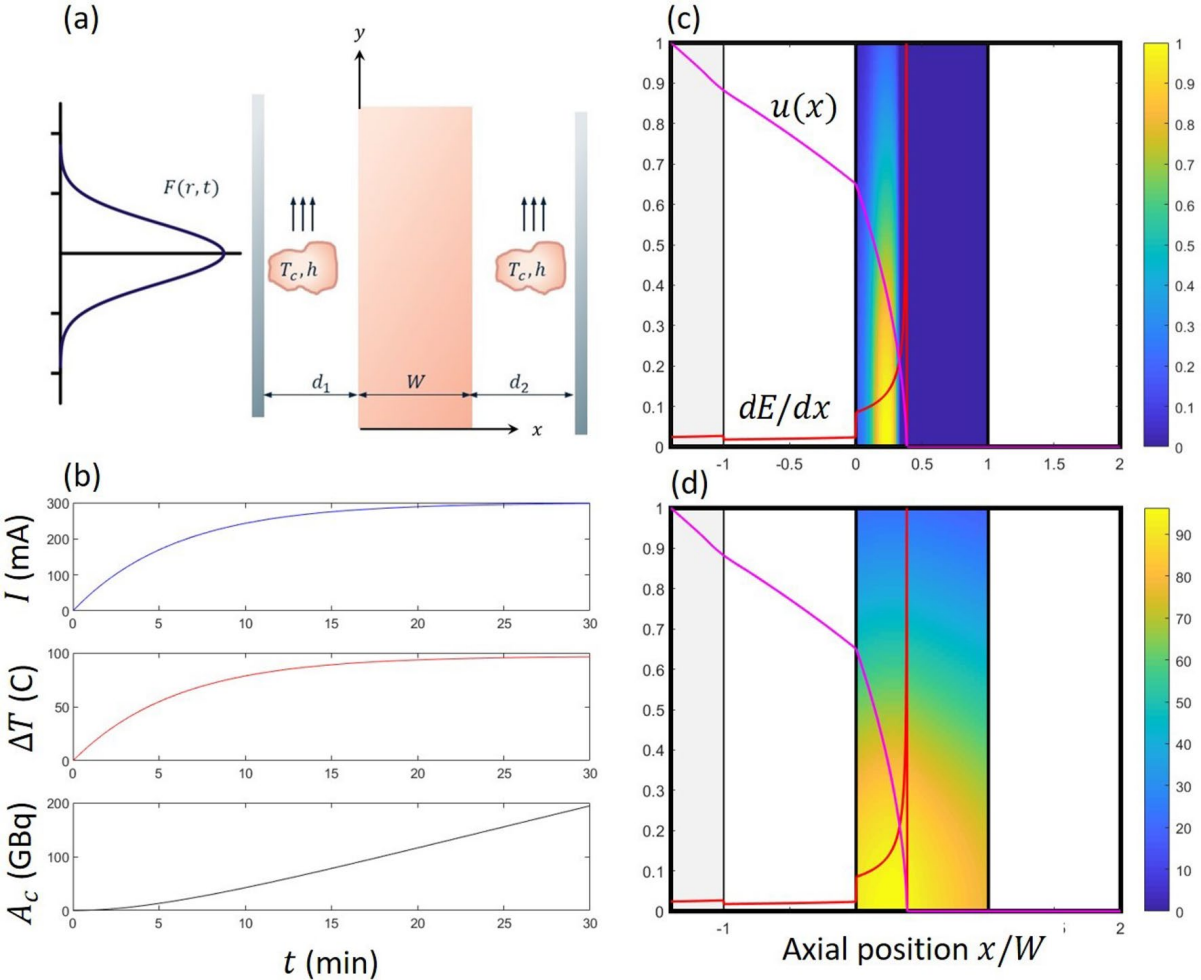
(**a**) Schematic of the updated geometry. The 40 MeV beam enters the target from the left through a 2 mm thick foil with $A=2.25\times 10^{32}$ m$^3$/s$^4$ and $B=1.46\times 10^{-13}$ s$^2$/m$^2$. The beam passes through the cooling water ($A=1.47\times 10^{32}$ m$^3$/s$^4$, $B=1.52\times 10^{-13}$ s$^2$/m$^2$) in a channel of thickness $d_1=5$ mm before coming to rest in a target of thickness $W=5$ mm ($A=1.20\times 10^{33}$ m$^3$/s$^4$, $B=2.71\times 10^{-14}$ s$^2$/m$^2$) and diameter of 50 mm. A second channel of thickness $d_2=5$ mm is located downstream and cools the other side of the target. A mass flowrate of 1 kg/s of water was set in each channel. The Gaussian curve, drawn in blue, represents the distribution in flux *F*(*r*, *t*) with $\omega /R=0.8$. (**b**) The conditions outlined by Tanguay et al.^9^ for the production of $^{100}Tc$ from $^{99m}Mo$. Here, the current of the beam *I* was slowly increased to 300 mA over the first 20 minutes of production. Following this it was held constant. In the two lower figures, the maximum temperature rise $\Delta T$ and the growth in $A_c$ as determined from the simulation. The heat transfer coefficient was set to $h=10,000$ W/m$^2$ K, thermal conductivity $k=132$ W/m K giving a Biot number $Bi=0.36$. (**c**) The color map represents the estimated distribution of *N*. Superimposed onto this is the solution for *u*, pink line, and energy dissipation *de*/*dx*, red line. (**d**) The color map represents the temperature distribution. The pink and red lines are defined as in (**c**). With a beam current of 300 $A$, we determine $F_o= 3.15\times 10^{24}$ particles/m$^2$ s using Eq. (5c). The intermediary steps to calculate *A* and *B* are given as S2 in the Supplemental Material.

The mathematical model posed for the $^{225}Ac$ solid target is general in nature and should, in principle, be applicable to other target materials. In this section we discuss the robustness and applicability of this approach to a wider range of targets. To do so we examine a case numerically where the range of the beam is shorter than the thickness of the width of the target. In particular we examine the production $^{99m}Tc$, which is perhaps the most widely used isotope for diagnostic purposes^9^. For this, we extended our original geometry (Fig. 1) to include an upstream foil which mechanically isolates the target from the cyclotron, and, a downstream plate which forms the target body; water flows in the upstream and downstream cavities, see Fig. 5a. We have allowed the temperature of the water stream to be heated by both the passage of the beam and by the cooling of the target. As such the water temperature varies slightly from the inlet to the outlet. A representative calculation for the $^{99m}Tc$ is shown in Fig. 5b–d where we highlight the beam position (Bethe Block), energy dissipation (Stopping Power), beam profile and range. We use the same operating conditions as outlined by Tanguay et al.^9^ where the beam current is increased slowly over the first 20 min of operation. We were able to reproduce the estimate of $A_c$ given by Tanguay et al. What is evident and somewhat expected is that the activity is concentrated within the range of the beam, see Fig. 5c. Further, we observe large temperature variations in the radial direction and attribute this to the width of the beam. The small temperature gradients in the *x*-direction are also anticipate as the Biot number is small. What we do find encouraging is that our numerical scheme is robust for both “beam-thin” and “beam-thick” conditions offering a novel means to understand transport phenomena in these targets.

## Summary

In this work we examined the production of $^{225}Ac$ in a solid target similar to that discussed by Roberston et al.^3^. After posing the model as a coupled system, where beam position and temperature field are coupled through density, our simulations indicate that the coupling was weak, and could be eliminated. We developed a numerical scheme to solve the uncoupled system and found that for all conditions relevant to Roberston et al.^3^, a small fraction of the total incoming energy of the beam was deposited into the target. As such, we found energy to decrease linearly and energy dissipation to be essentially constant, in each layer of the composite target. This finding allowed us to extend widely-accept work by^1^ and advance asymptotic solution in the beam-thin limit. These solutions agreed well with the simulations and set the theoretical framework to interpret the findings. We examined in the last portion of this work transient behavior, created by modulating the beam in a sequence of on-off cycles. Here we demonstrate that we can cycle the temperature of the target, from ambient to a prescribed set-point, and then back to ambient. As the half-life of the isotope is long in comparison cycle time, little decay occurs, allowing us to reach a desired activity by repeating the cyclic pulses

### Supplementary Information

Supplementary Information.

## Data Availability

The datasets used and/or analysed during the current study available from the corresponding author on reasonable request.
