# Supplementary material for: Estimating 225Ac yield in thorium metal targets
Source: Sci Rep. 2023 Sep 22;13:15848. doi: 10.1038/s41598-023-41687-0 (PMC10516867; doi:10.1038/s41598-023-41687-0)
Supplement: Supplementary file 1 — Supplementary Information. [file 41598_2023_41687_MOESM1_ESM.pdf]

Supplemental Material - Optimizing Isotope Production for Radiotherapy: Estimating ^{225}Ac Yield in Thorium Metal Targets

M Rahmani[1,*] and D. M. Martinez[1]

[1]University of British Columbia, Department of Chemical and Biological Engineering, Vancouver, BC, V6T 1Z4, Canada
[*]mona.rahmani@ubc.ca

ABSTRACT

Enclosed is the supplementary material for this publication

S1 Derivation of Equation 19

We begin by restating the governing equation for convenience:

$$\frac{1}{\bar{r}}\frac{\mathrm{d}}{\mathrm{d}\bar{r}}\left(\bar{r}\frac{\mathrm{d}\theta}{\mathrm{d}\bar{r}}\right) - 2Bi\theta + 1 - (\frac{\bar{r}}{\bar{\omega}})^2 + \frac{1}{2}(\frac{\bar{r}}{\bar{\omega}})^4 = 0 \tag{1}$$

When integrated, we find that

$$\theta = -\frac{\left(Bi\,\bar{\omega}^2 - Bi - 4\right)}{Bi^2\bar{\omega}^4\sqrt{-2Bi}}\frac{J_0\left(\sqrt{-2Bi}\,\bar{r}\right)}{J_1\left(\sqrt{-2Bi}\right)} + \frac{16+\left(2\,\bar{\omega}^4 - 2\,\bar{\omega}^2\bar{r}^2 + \bar{r}^4\right)Bi^2 + \left(-4\,\bar{\omega}^2 + 8\,\bar{r}^2\right)Bi}{4\,Bi^3\,\bar{\omega}^4} \tag{2}$$

where J_i are Bessel functions. Using the following definition of a modified Bessel function,

$$J_0\left(\sqrt{-2Bi}\,\bar{r}\right) = J_0\left(i\sqrt{2Bi}\,\bar{r}\right) = I_0\left(\sqrt{2Bi}\,\bar{r}\right) \quad \& \quad J_1\left(\sqrt{-2Bi}\right) = J_1\left(i\sqrt{2Bi}\right) = iI_1\left(\sqrt{2Bi}\right) \tag{3}$$

Eq. 1 reduces to

$$\theta = -\frac{\left(Bi\,\bar{\omega}^2 - Bi - 4\right)}{Bi^2\bar{\omega}^4\sqrt{2Bi}}\frac{I_0\left(\sqrt{2Bi}\,\bar{r}\right)}{i^2 I_1\left(\sqrt{2Bi}\right)} + \frac{16+\left(2\,\bar{\omega}^4 - 2\,\bar{\omega}^2\bar{r}^2 + \bar{r}^4\right)Bi^2 + \left(-4\,\bar{\omega}^2 + 8\,\bar{r}^2\right)Bi}{4\,Bi^3\,\bar{\omega}^4} \tag{4}$$

As $i^2 = -1$, we report the temperature distribution as

$$\theta = +\frac{\left(Bi\,\bar{\omega}^2 - Bi - 4\right)}{Bi^2\bar{\omega}^4\sqrt{2Bi}}\frac{I_0\left(\sqrt{2Bi}\,\bar{r}\right)}{I_1\left(\sqrt{2Bi}\right)} + \frac{16+\left(2\,\bar{\omega}^4 - 2\,\bar{\omega}^2\bar{r}^2 + \bar{r}^4\right)Bi^2 + \left(-4\,\bar{\omega}^2 + 8\,\bar{r}^2\right)Bi}{4\,Bi^3\,\bar{\omega}^4} \tag{5}$$

	Th	Inconel 600	Water	Mo
Number of electrons z	90	28	10	42
Electron density $n \times 10^{30}$ (m^{-3})	2.7	2.6	0.3	2.7
Electron mass $m_e \times 10^{-31}$ (kg)	9.1	9.1	9.1	9.1
Electron charge $e \times 10^{-19}$ (C)	1.6	1.6	1.6	1.6
Vacuum permittivity $\varepsilon_0 \times 10^{-12}$ (C^2 s^2/Kg m^3)	8.9	8.9	8.9	8.9
Ionization potential $e \times 10^{-17}$ (C)	1.4	4.1	1.2	6.7
Particle mass $m_p \times 10^{-27}$ (C)	1.67	1.67	1.67	1.67
A $\times 10^{33}$ (m^3/s^4)	1.20	1.13	0.15	1.20
B $\times 10^{-14}$ (s^2/m^2)	1.3	4.4	15.2	2.7

Supplementary Table 1. A listing of the parameters used to determine A and B

S2 Determining A and B for the Bethe Equation

Following Grimes *et al.*[1], we set

$$A = \frac{4\pi n z^2}{m_e m_p}\left(\frac{e^2}{4\pi\varepsilon_0}\right)^2 \qquad B = \frac{2m_e}{I} \tag{6}$$

where the parameters are defined in Table 1 for materials used in the simulations.

References

1. Grimes, D. R., Warren, D. R. & Partridge, M. An approximate analytical solution of the Bethe equation for charged particles in the radiotherapeutic energy range. *Scientific Reports* **7**, 9781 (2017).
